# High-Intensity Focused Ultrasound in Dentistry: A Literature Review

**DOI:** 10.1016/j.identj.2024.02.004

**Published:** 2024-04-11

**Authors:** Minh Dien Tran, Hien Ngo, Amr Fawzy

**Affiliations:** Restorative Dentistry/Dental Biomaterials Research Group, UWA Dental School/The University of Western Australia, Perth, Australia

**Keywords:** HIFU, Dentine remineralisation, Smear layer removal, Peri-implantitis, Biofilm removal destruction, Drug delivery

## Abstract

Although high-intensity focused ultrasound (HIFU) has been applied widely in medicine, utilising its non-invasive dual ablation and thermal coagulation properties, its application in dentistry has primarily remained in the research phase, predominantly in *in vitro* studies. Nonetheless, there has been a consistent increase in the number of publications on this subject in recent decades, focusing on areas such as remineralisation of dentine surfaces, removal of smear layers, drug delivery, and microbial elimination. The number of advantages HIFU can offer, such as its non-surgical nature, absence of ionising radiation, lack of residue, and absence of aerosols, is driving this upward trend, indicating the potential for HIFU in clinical dentistry and ongoing efforts towards developing HIFU-based devices for routine dental use. This succinct review aims to outline the historical context, operational mechanisms of HIFU, summarise recent dental research, and provide a forward-looking perspective on the role of HIFU in modern clinical dentistry.

## History

Although it has been known that the first high-intensity focussed ultrasound (HIFU) biological effect was discovered during World War II, in which submarine navigation using HIFU found heating up and killing fish,^1^ the effects of “high-frequency sound-waves of great intensity” were described as early as 1928 by Wood and Loomis.^2^ In this very first publication, the thermodynamic effects of 700 kHz soundwaves generated by piezo-electric oscillator exhibited a disruptive action that destroyed red blood cells, and killed small fish and frogs.^2^ The therapeutic significance of HIFU in medicine was first studied in 1942 by Lynn et al.^3^ In their experiment, the application of HIFU led to significant damage with clear boundaries in beef livers. However, when HIFU was applied to animal brains, it resulted in unintended and severe injuries to the skin and underlying subcutaneous tissues.^3^ Lynn et al^3^ indicated that technical improvement was mandatory before clinical application was possible in brain therapy.

The development of therapeutic HIFU did not accelerate until late 1950s and early 1960s, when ultrasound guidance systems were integrated to pinpoint focal points. During this period, noteworthy studies were conducted by Fry et al^4^ on the animal central nervous system; however, this guiding technology had limited accuracy and did not provide instant temperature feedback.^1^, ^5^ The introduction of magnetic resonance imaging guidance systems in the 1980s helped to overcome these limitations and allowed HIFU to be used on sensitive structures such as the brain.^1^, ^5^ Nowadays, HIFU is applied widely in medicine in areas ranging from prostate cancer treatment to Parkinson disease to neuropathic pain^5^ as a non-invasive and non-ionising therapeutic modality.

## HIFU working mechanisms

The main differences between diagnostic ultrasound and HIFU are the intensity, frequency, and focus of the ultrasound waves.^6^ Diagnostic ultrasound has an intensity below 0.1 W/cm^2^, and the frequency ranges from between 3 and 5 MHz for deeper structures (abdominal) to between 5 and 10 MHz for superficial organs such as muscles and breasts to between 10 to 30 MHz for the skin and eyes.^6^ These diagnostic ultrasound beams generally travel at 90° to the transducer face, which is convex, creating a fan-out field of view. These high-frequency, low-intensity, and nonfocussed soundwaves do not cause physical or thermal damage to living tissue.^6^

In HIFU, the sound intensity varies between 0.125 and 10,000 W/cm^2^, depending on the intended effects. The lower range (0.125–3 W/cm^2^) induces mechanical effects at a cellular level, whilst the higher intensity (100–10,000 W/cm^2^) is used for tissue ablation.^7^ The HIFU beams typically have a frequency between 0.2 and 5 MHz; however, the lower end of the ultrasound frequency spectrum of 27 kHz has been observed in certain studies^8^^,^^9^ to maximise ablation whilst reducing hyperthermal effects.^10^ The beams are highly focused, using either geometric/mechanical or electronic focusing mechanisms.^7^

According to Kalamarz et al,^11^ the mechanical focussing is achieved with the concave curvature of the ultrasound transducer surface and the refraction of the beams (Figure). Due to the higher velocity of the ultrasound inside the transducer compared with the tissues, the ultrasound beam direction changes at the interface according to the laws of refraction and the focal point is calculated using trigonometric relationships.^11^ In the electronic focussing method used in phased array transducers, the individual time delay for each transducer element is processed based on the distance to the focal point and soundwave velocity. The electrical impulses are then combined into one electrical pulse, creating focusing echoes^11^ (Figure).

**Figure:**
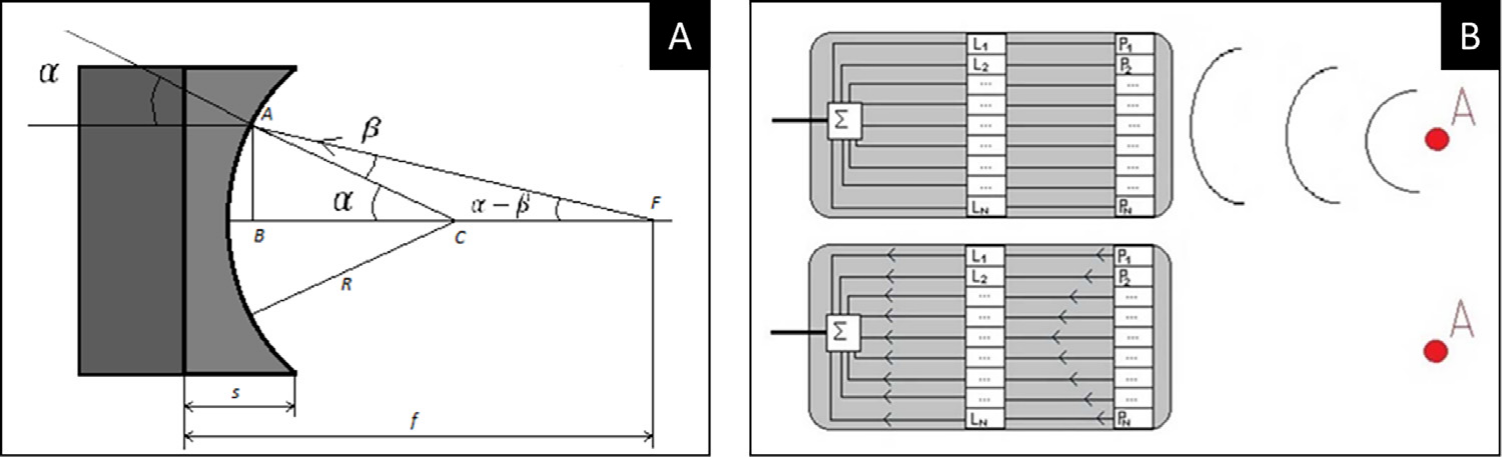
Principle of geometric/mechanical focusing vs electronic high-intensity focused ultrasound (HIFU) mechanisms. **A**, Geometric focusing: focal point (f) is calculated using the formula AB = R sin α = f sin (α – β) where R = transducer surface curvature radius. **B**, Electronic focusing mechanism using time delay line (L) to generate a combined single electrical impulse from various elements, resulting in focusing echoes. Courtesy of Kalamarz et al.^11^

One benefit of electronic focusing is its capability to adjust the focal point position without needing to physically move the transducers; this is known as electronic steering.^1^^,^^7^ There are 2 fundamental physical impacts of HIFU on the target tissues: hyperthermia and ablation.

As the HIFU waves propagate through the media, the pressure fluctuation creates a microscopic shearing motion of the particles, resulting in friction heat. This hyperthermic effect is a function of its frequency, intensity, and application time. This relationship is expressed in the equation^10^:

$$q=0.052f^{1.1}It$$

where *q* is the heat generated in W/cm^3^, *I* is the intensity in W/cm^2^, *f* is the sound frequency in MHz, and *t* is the exposure time in second.

This formula suggests employing higher frequencies when aiming for thermal coagulation and lower frequencies when this biological effect is undesirable. According to Ter Haar and Coussios,^12^ the frequency range of 0.5 to 8 MHz is used in most clinical HIFU appliances.

The HIFU cavitation is the result of rapid sound pressure changes within the medium, which lead to the formation and collapse of the bubbles. This phenomenon can cause mechanical damage to the target tissues, or ablation, which is represented mathematically by the mechanical index (MI). This parameter is a function of the peak negative pressure and the central frequency of the HIFU^10^:

$$MI\;=\;Pra\;/\;\sqrt{fc}$$

where MI is the mechanical index, *P_ra_* is the peak negative pressure in MPa, and *fc* is the central frequency in MHz.

The nature of HIFU waves can be represented using the hydrophone and recording device.

According to Qu et al,^10^ the value of MI must be greater than 1.9 to have any bioeffects, and this value can be increased with lower frequency. This value has been approved by the US Food and Drug Administration as the highest threshold in diagnostic ultrasound.^10^

In summary, both HIFU thermal and mechanical impacts are greater when its intensity is increased, whereas reducing the frequency will lead to lessened thermal but strengthened cavitation effects.

As HIFU waves travel through transmission media, their intensity is lost depending on the distance and the attenuation coefficient of the media,^12^ which can be defined as the reduction in amplitude of the ultrasound beam and expressed in the equation *I = I_0_e^−µx^*, where *I_0_* is the incident intensity, *x* is the distance, and *µ* is the attenuation coefficient.^12^

The acoustic attenuation coefficient is tissue-specific and frequency-dependent,^12^ as seen in the equation *µ = af^b^*, where *a* and *b* are tissue-specific constants and *f* is the frequency.

This attenuation coefficient varies widely in different biological tissues, and water exhibits tremendously lower value compared to other tissues, prompting the use of water-based media for ultrasound transmission. At 1 MHz, the attenuation coefficient for water was 0.0022 compared to 6.9 for cortical bone, 9.94 for trabecular bone, 80 for dentine, 120 for enamel, 1.57 for connective tissue, 0.54 on average for soft tissue, 0.2 for blood, 1.09 for muscle, and 4.7 for tendon.^13^

## Dental HIFU

Despite HIFU's popularity in medicine, its application in the dental field is still in research stage; however, research in this area has steadily increased in the past decade, particularly in dental biomaterials. A Google Scholar search using the key words “dentistry AND high intensity focused ultrasound” from 2010 to 2020 resulted in a 100% increase in published articles in this field, with a steady upward trend, and the majority of these articles have involved dentine and resin bonding.

A search of literature on 22 September 2021 with the key words “dental HIFU” using OneSearch search engine resulted in 292 published articles. A total number of 13 significant dental-related research articles were selected, based on titles and abstracts, for review. Their author(s), year of publication, aims, and results and the significance of 8 most dentally relevant papers are summarised in Table 1 and Table 2. Earlier studies utilised a setup comprising a function generator that transmitted sinusoidal signals at a designated frequency to a voltage amplifier. Subsequently, HIFU was produced via a geometric transducer upon receiving these waveform currents.^9^^,^^14^ In more recent research, a commercially available HIFU generator had the capability to generate amplified signals^15^, ^16^, ^17^, ^18^ at different frequencies, as determined by the operator. Additional progress includes the development of phase array transducers and the significant reduced diameter of the transducers, from 80 mm in 2009^8^ to 20.2 mm in 2021^15^ (Table 2), thereby enabling the potential use of transducers for intraoral applications.

**Table 1:** Summary of published dental-related high-intensity focussed ultrasound (HIFU) studies.

Year, type	Author(s)	Aims	Results	Significance
2009, in vitro	Ohl et al^8^	Delivery of antibacterial nanoparticles into dentinal tubules	Penetration of antibacterial chitosan-sodium tripolyphosphate nanoparticles up to 1000 microns into dentine tubules	Drug delivery system, root canal disinfection
2009, in vitro	Bigelow^14^	*E coli* biofilm destruction	Killed *E coli* biofilm on microscope slides	Noninvasive bacterial biofilm removal and destruction
2009, in vitro	Shrestha et al^9^	Delivery of nanoparticles into dentinal tubules	Penetration of chitosan polymer nanoparticles up to 1000 microns deep into dentinal tubules	Drug delivery system into dentine
2013, in vitro	Iqbal et al^19^	Bacterial biofilm and planktonic suspension destruction	Removed and killed *E faecalis* bacterial suspension and biofilms on petri dish	Root canal disinfection
2019, in vitro	Daood et al^16^	HIFU on cementum	Smear layer removal, surface smoothened	Periodontal debridement
2019, in vitro	Fawzy et al^18^	Dentine surface modification	Removal of smear layer, opening of dentinal tubules seen in acid etching	Potential acid etching alternative
2020, in vitro	Daood et al^17^	Dentine remineralisation	Remineralisation of dentine with hydroxyapatite nanorods	Caries therapies
2021, in vitro	Daood et al^15^	Dentine biofilm growth inhibition	Inhibited *S mutans* biofilm growth on dentine collagen and associated matrix metalloproteinases	Caries control

**Table 2:** Summary of the materials and methods of the published dental-related high-intensity focussed ultrasound (HIFU) studies.

Author, year, type of research	Transducer type	Diameter	Focal point	Frequency, voltage, intensity, pressure	Methodology
Ohl,^8^ 2009, in vitro	Piezo ceramic	80 mm	80 mm	27 kHz, 127 V	Exposed dental root dentinal tubules submerged in 80–120 nm chitosan nanoparticle suspension in a test tube which was immersed in water tank; FESEM and EDX analysis
Bigelow, ^14^ 2009, in vitro	Sonic Concepts	70 mm	63 mm	1.0 MHz, various peak negative pressure between 0 and 7.6 MPa	*E. coli* biofilm on microscope slides kept in saline plastic bag which was immersed in degassed water; Raster scan 750 micron step size, 30 s between steps for 3 h 40 ft in water; CFUs evaluation
Shrestha, ^9^ 2009, in vitro	Unspecified disc ceramic	8 mm	15 mm	27 kHz, 126 V	Chitosan polymer and tripolyphosphate nanoparticles (80–120 nm) in acetic solution; penetration into dentinal tubules measured by FESEM and EDS
Iqbal, ^19^ 2013, in vitro	Piezo-ceramic (H-115 Sonic Concepts) circuited to linear voltage amplifier and signal generator	64 mm	59.97 mm focal point and 50.65 mm focal depth	0.25 MHz; 120 Vpp continuous sinusoidal HIFU waves; maximal 10 bar pressure	*E faecalis* planktonic and biofilm on glass petri dish; 30, 60, and 120 s HIFU exposure in water; SYTO9/PI stain; CFUs, SEM, and CLSM evaluation
Daood,^16^ 2019, in vitro	Piezo-ceramic (H-115 Sonic Concepts) circuited to linear voltage amplifier and signal generator	64 mm	59.97 mm focal point and 50.65 mm focal depth	0.25 MHz; 120 Vpp continuous sinusoidal HIFU waves; maximal 10 bar pressure	Cementum discs exposed to HIFU at 15, 30, and 60 in; SEM for surface characterisation; AFM for micro-roughness and stiffness; CLSM, fluorescent light microscopy, and Raman for microphage proliferation on cementum
Fawzy,^19^ 2019, in vitro	Piezo-ceramic (H-115 Sonic Concepts) circuited to linear voltage amplifier and signal generator	64 mm	59.97 mm focal point and 50.65 mm focal depth	0.25 MHz; 120 Vpp continuous sinusoidal HIFU waves; maximal 10 bar pressure	Coronal dentine surface (discs) treated with HIFU in degassed water for 60–120 s; specimens compared with the controls (35% phosphoric acid etch for 15 s); resin–dentine bonding in the 2 groups (Singlebond-Filtek Z350) was also compared using AFM, SEM, TEM, nano-indentation, and Raman analysis
Daood,^17^ 2020, in vitro	Piezo-ceramic, bowl-shaped, transducer (H-115, Sonic Concepts)	64 mm	59.97 mm focal point and 50.65 mm focal depth	0.25 Mhz; 184 mV/MPa; maximal 10 bar pressure	Human dentine discs were etched, immersed in hydroxyapatite solution, and exposed to HIFU for 5, 10, and 20 s, and then stored in artificial saliva for 1 mo; EDS, SEM, AFM, Raman spectroscopy, and TEM data were collected and compared with control (etched dentine, immersed in distilled water and stored in saliva)
Daood,^15^ 2021, in vitro	Piezo-ceramic bowl-shaped transducer (TQN20-20C30, Siansonic Technology Ltd)	20.2 mm	18.4 mm	1 ± 4% MHz; 184 mV/MPa HIFU waves; maximum pressure of 10 bar	Human dentine discs were exposed to HIFU for 5, 10, and 20 s; XPS to determine surface elements; Raman spectroscopy for collagen orientation mapping and biochemical analysis; human dental pulpal cells were cultured on dentine surface, exposed to HIFU, and collected for calcium measurement; similar protocol was used for macrophage and *S mutans* biofilms

AFM, Atomic Force Microscopy; CLSM, Confocal Laser Scanning Microscopy; CFU, Colony-Forming Unit; EDS, Energy Dispersive X-ray Spectroscopy; FESEM, Field Emission Scanning Electron Microscopy; PI, Propidium Iodide; SEM, Scanning Electron Microscopy; TEM, Transmission Electron Microscopy; XPS, X-ray Photoelectron Spectroscopy.

Regarding antimicrobial properties, HIFU has shown the ability to (1) enhance the penetration of antibacterials up to 1000 µm into dentinal tubules^8^^,^^9^; (2) inhibit *Streptococcus mutans* growth on dentine^15^; and (3) remove and destroy *Escherichia coli* and *Enterococcus faecalis* biofilms.^14^^,^^19^ The smear layer removal^16^^,^^18^ and remineralisation^17^ effects of HIFU on dentine could be investigated further for caries control and dental adhesion.

The results of these studies have confirmed the potential of HIFU in many aspects of dentistry, along with its relatively low cost and uncomplicated setup. However, the in vitro studies reviewed have also pointed out the difficulty of bringing HIFU research to the next level in animal studies and human trials. In addition, this literature review also showed the shortage of studies of HIFU on dental implants, particularly its ability to destroy and remove implant-attached bacterial biofilms, which are the principal pathogenesis factor in peri-implantitis.^20^, ^21^, ^22^, ^23^, ^24^, ^25^, ^26^, ^27^, ^28^, ^29^

In the absence of perfect debridement technique for biofilms attached to implants,^21^, ^25^ HIFU offers a promising new research field with its distinctive features of being free from aerosols and non-ionizing residues.

## Conclusions

HIFU has demonstrated biological effects as early as 1928 and has been widely utilised in medicine as a noninvasive, nonaerosol, nonionising, and residue-free therapeutic tool, employing both ablation and hyperthermia effects. However, research into its dental applications spans less than 3 decades and primarily comprises in vitro studies. This narrative literature review illustrates the potential utilisation of HIFU in various dental disciplines, including caries control, dentine bonding, and endodontics. Furthermore, exploring HIFU's ablation properties in dental implant debridement is essential for managing periimplant diseases. The challenges in advancing HIFU research to the next phase, involving animal studies and human trials, primarily revolve around technical rather than conceptual obstacles.

The speculated causes for these slow advancements include the lack of commercially available intraoral HIFU transducers designed to mimic dental handpieces as well as the absence of suitable transmission media and their containment for oral cavity use. Moreover, the intricate morphology of dental implants, their high expense, and the dominance of anaerobic microorganisms related to peri-implant diseases contribute to further complexities in advancing HIFU research in dentistry overall, particularly concerning implants. Despite these challenges, the advancement of smaller HIFU transducers driven by growing research interest, currently sized at 4 to 6 mm (Siansonic),^30^ suggests that intraoral HIFU could become clinically accessible in the future.

## Author contributions

The authors confirm contributions to the paper as follows. Study conception and design: Minh Dien Tran, Amr Fawzy, and Hien Ngo. Data collection: Minh Dien Tran. Analysis and interpretation of results: Minh Dien Tran. Draft manuscript preparation: Minh Dien Tran, Hien Ngo, and Amr Fawzy. All authors reviewed the results and approved the final version of the manuscript.

## Conflict of interest

None disclosed.
